# The effect of chronic lithium treatment on hippocampal progenitor cells: Transcriptomic analysis and systems pharmacology

**DOI:** 10.1002/brb3.3215

**Published:** 2023-08-08

**Authors:** Mina Jahandideh, Erfan Ebrahimi, Mohammad Hosein Farzaei, Ebrahim Barzegari

**Affiliations:** ^1^ Medical Biology Research Center Health Technology Institute Kermanshah University of Medical Sciences Kermanshah Iran; ^2^ Student Research Committee Kermanshah University of Medical Sciences Kermanshah Iran; ^3^ Pharmaceutical Sciences Research Center, Health Institute Kermanshah University of Medical Sciences Kermanshah Iran

**Keywords:** bipolar disorder, hippocampal gyrus, lithium, pharmacogenomics, RNA‐sequence analysis

## Abstract

**Objective:**

To identify the genomics underpinning the increased volume of the hippocampus after long‐term administration of lithium (Li) in bipolar disorder patients, hypothesizing the possible contribution of cell growth and differentiation pathways to this complication.

**Methods:**

RNA‐seq profiles of four samples of hippocampal progenitor cells chronically treated with a high dose of Li and three samples chronically treated with the therapeutic dose were retrieved from NCBI‐GEO. The raw data underwent filtration, quality control, expression fold change, adjusted significance, functional enrichment, and pharmacogenomic analyses.

**Results:**

CCND1, LOXL2, and PRNP were identified as the genes involved in the drug response and the chronic effects of Li in the hippocampal cells. GSK‐3β was also a hub in the pharmacogenomic network of Li. In addition, ZMPSTE24 and DHX35 were identified as the important genes in lithium therapy.

**Conclusions:**

As shown by gene ontology results, these findings conclude that lithium may increase the size of the hippocampus in bipolar patients by stimulating the generation of new neurons and promoting their differentiation into neuroblasts, neurons, or microglia.

## Graphical abstract

The research reports the application of a network pharmacology approach to identify the pharmacogenomics underpinning the complications presenting after long‐term lithium medication of bipolar disorder patients, hypothesizing the possible contribution of cell growth and differentiation biochemical pathways to this complication. Our findings from transcriptomic data analyses indicate that lithium may stimulate the generation of new neurons and promote their differentiation into neuroblasts, neurons or microglia in the hippocampus of bipolar patients by modulating some genes' expression. This work is thus of high significance and holds substantial potential as the background for better identifying the lithium pharmacological genomic network.

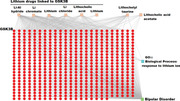

## 1 INTRODUCTION

Bipolar disorder (BPD) and schizophrenia are severe psychiatric illnesses with a combined prevalence of 4% of the world population (Clay et al., 2011). The monovalent cation lithium (Li) is the first‐line treatment for BPD and the most effective in maintaining and preventing episodes of mood swings. The drug is prescribed lifelong when clinically appropriate (Sköld et al., 2021). Li is thought to exert its therapeutic and mood‐stabilizing effects by acting on cellular targets, specifically through the inhibition of glycogen synthase kinase 3 (GSK‐3), and modulating the molecular functions in neuronal pathways (Malhi et al., 2013). Nevertheless, it still remains difficult to elucidate the exact pathophysiology differentiating BPD from its associated diseases and the role of lithium therapy in this disorder (Akkouh et al., 2020).

Built on systems bioinformatics and histology, the constantly evolving science of network pharmacology and its resultant technologies integrate drugs’ data and large molecular information networks in a high‐throughput manner to directly identify drugs and disease targets and to understand the mechanisms and pathways connecting the drug, the disease, and the target together (Hao da & Xiao, 2014; Lai et al., 2020). This field also combines network biology with polypharmacology to uncover the basic medicinal roles of drugs, their side effects, and the poor efficacy of highly selective single‐target drugs (Guo et al., 2019; Sharma, Singla, et al., 2022).

As Li has multiple molecular targets in the body, its therapeutic role in BPD may have aspects other than inhibiting GSK‐3 (Kerr et al., 2018; Pisanu et al., 2018). Moreover, GSK‐3 inhibition has shown molecular consequences such as the chronic side complications observed in the hippocampus of BPD patients (Kerr et al., 2018; Palmos et al., 2021). Brain images have shown that lithium used as a BPD treatment accumulates in the hippocampus tissue over time and causes the hippocampal volume to increase (Hajek et al., 2012; Hibar et al., 2016). In 2021, Palmos et al. (2021) tested the chronic effect of low or high doses of lithium on human hippocampal progenitor cells and used immunocytochemistry to investigate the effect of Li on neurogenesis. RNA sequencing and gene set enrichment were used to show whether the genes affected by the drug are involved in regulating the volume of specific layers of the dentate gyrus. The current study intends to apply a systems pharmacology approach to investigate the interactions of Li with its molecular targets in progenitor cell models of the human BPD‐affected hippocampus so as to reveal other aspects of the communication among the disease, the drug, and the targets.

## 2 METHODS

### 2.1 Data retrieval and analysis

The expression data of the cells composing the hippocampal neuronal tissue, before and after being treated with lithium, were retrieved from the NCBI Gene Expression Omnibus (GEO) (RRID:SCR_005012) database (accession code GSE184930). This expression profile reports the application of two different doses of lithium on hippocampal progenitor cells and compares them with the control (no drug). The low dose (0.75 mM) represented the blood concentration of Li in bipolar patients, whereas the high dose (2.25 mM) represented the cumulative effect of the drug. In line with the recent clinical longitudinal investigation (Spielberg et al., 2018) and like in other studies (De‐Paula & Forlenza, 2021; De‐Paula et al., 2016), the low and high doses were continuously applied to the samples for 15 days to reflect the chronic effect of Li. However, as the chronic lithium complications such as the enlarged hippocampus might be more likely in high doses than in low doses (Palmos et al., 2021), the gene expression effects observed in the high dose were interpreted as the long‐term outcomes of the drug and its resulting cumulative effects. RNA‐seq was done in paired‐end mode. Quantification was performed with the Kallisto (RRID:SCR_016582) software and normalization with DESeq2 (RRID:SCR_015687). In the present study, raw count files obtained from Kallisto were downloaded and underwent RNA‐seq analysis.

To reduce the error in identifying important genes, unexpressed or underexpressed genes were removed from the analysis. There are several methods for this filtering out. Normally, a number can be specified as the minimum number of reads of a gene to be included in the analysis. If replications exist in any test group, a number can be determined as the minimum count per million (CPM) in a minimum number of samples. In this study, the minimum number of reads required to include a gene was set to 10, and the minimum number of copies per million was determined to be 0.5 in at least three samples.

RNA‐seq data analysis was performed with the Voom/LIMMA (RRID:SCR_010943) algorithm, a code written in R (RRID:SCR_001905), and implemented using DeGust (RRID:SCR_001878) (Powell, 2019). Gene interaction network was drawn and analyzed in Cytoscape (RRID:SCR_003032). Functional data were obtained from GeneCards (https://www.genecards.org) (RRID:SCR_002773). A *p*‐value less than .05 designated statistical significance.

### 2.2 Statistical analysis

The parameter to decide whether genes are important is false discovery rate (FDR), which is the *p*‐value adjusted by the Benjamini–Hochberg method. It indicates the significance of the gene expression changes among different groups. FDR < 0.05 was considered significant. The expression fold change (FC) shows the multiplication of the expression of a specific gene compared to its average expression or compared to the control. It is typically shown logarithmically as logFC. Positive logFC values indicate increased expression of the desired gene, whereas negative values designate its decreased expression.

## 3 RESULTS

### 3.1 Filtering out underexpressed genes

By applying the CPM filters to exclude low‐expression genes in the samples, 12,792 genes were selected to enter the analysis stage.

### 3.2 Gene count data quality control

**FIGURE 1:**
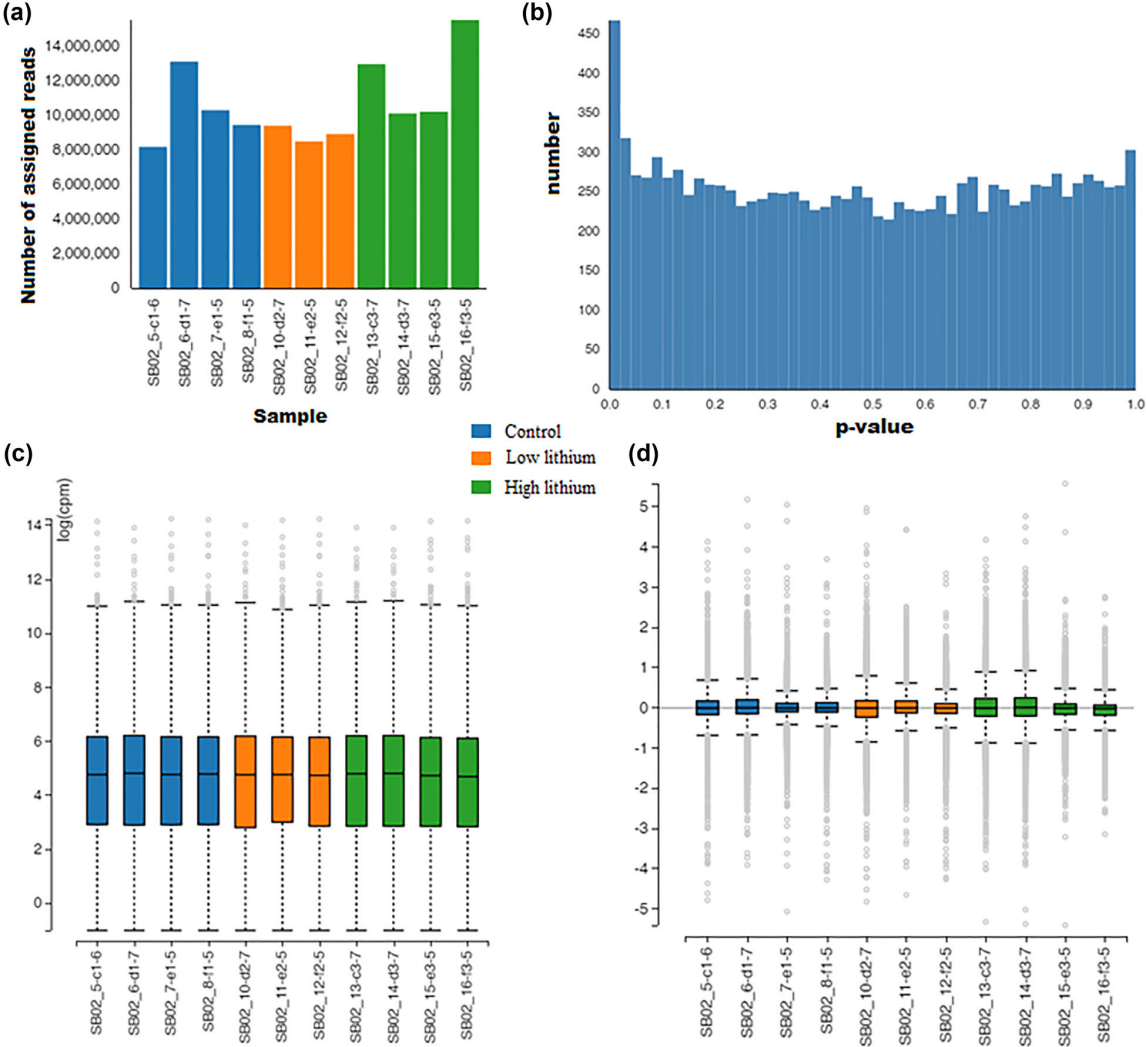
Data quality control of the selected genes for the RNA‐seq transcriptomics of lithium treatment samples of hippocampal progenitor cells: (A) library sizes (samples’ gene counts); (B) *p*‐value histogram showing the distribution of probabilities among the studied genes; (C) expression boxplot showing the distribution of (the log of) gene counts for the samples; (D) relative log expression, showing the distribution of (the log of) gene expression in the samples.

Figure 1 illustrates the RNA‐seq transcriptomic data quality assessment diagrams. Figure 1a shows the number of gene reads for each sample. As can be seen, none of the samples was an outlier with regard to the other ones. In Figure 1b, the frequency distribution of *p*‐values for the included genes has been depicted. Figure 1c shows the distribution of gene read count values (as the logarithm of the CPM; logCPM) for the studied samples in a boxplot and helps to see if the samples are homogeneous and comparable. The equal median values and quartiles among the samples indicate that they are cross‐comparable. Figure 1d presents a similar comparison but in terms of the logarithm of the expression level in the samples. This boxplot again confirms the comparability of the data.

### 3.3 Threesome comparison

**FIGURE 2:**
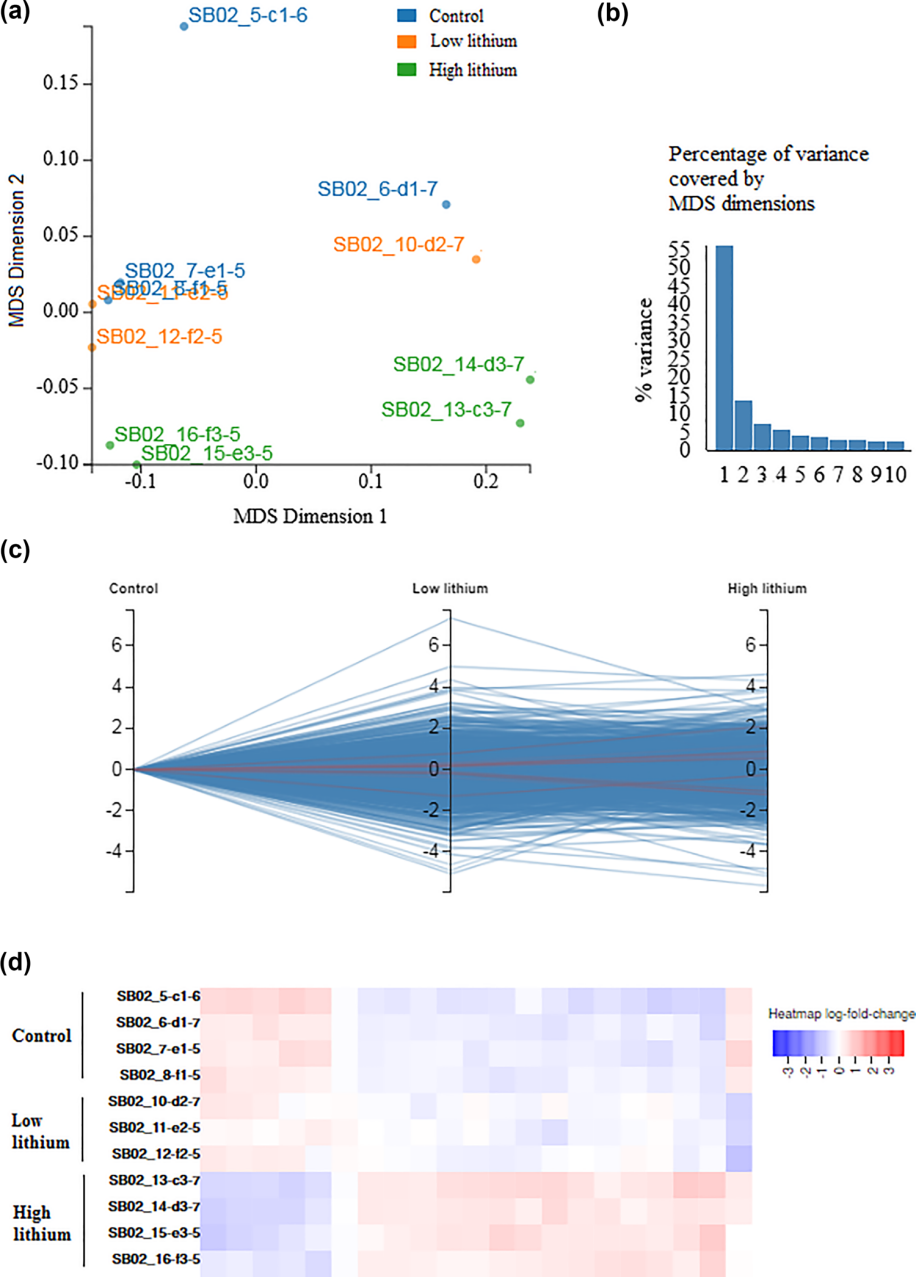
Threesome comparison: (a) multidimensional scaling (MDS) plot; (b) percent of variance covered by MDS dimensions; (c) parallel coordinates diagram; (d) heatmap for the genes with significant expression changes among the control, low‐dose, and high‐dose lithium samples.

The multidimensional scaling (MDS) diagram plots the distances between the major elements extracted from the study samples in a manner like the principal components analysis. The ideal situation is that the samples of one group are close to each other and far from the samples of other groups. As can be seen in Figure 2a, the samples in each of the control, low lithium, and high lithium groups are almost close to each other, with each group being differentiable from the other two groups. Furthermore, a high percentage of data dispersion (variance) is covered by the first component of the MDS (Figure 2b). These observations confirm the high quality of the data and their validity for threesome analysis.

The parallel coordinates diagram shows how logFC changes from one group to another. Each line in the plot represents a gene, with significantly changed ones marked with red lines. This graph gives an overview of the number of significant genes and how they change between the study groups. According to the parallel coordinates in Figure 2c, a number of genes have significant expression changes. Details of the expression for these genes are presented in Figure 2d and Table 1.

**TABLE 1:** Genes with significant differential expression among low‐dose Li, high‐dose Li, and control samples.

Gene	Control	Low lithium	High lithium	FDR	AveExpr	*p* Value
CD74	0	−0.1359	−1.03943	0.022483	8.941747	7.95E − 06
HLA‐DMA	0	−0.21417	−1.20494	0.022483	5.011998	3.04E − 06
L1CAM	0	0.781083	2.125241	0.022483	3.508505	8.79E − 06
PDLIM1	0	0.183111	0.885089	0.022483	6.493979	5.9E − 06
ZMPSTE24	0	−1.29206	−0.25703	0.022483	6.257451	4.44E − 06
SSR3	0	0.190003	0.546547	0.02348	8.591011	1.1E − 05
PMEPA1	0	0.314441	0.721328	0.036416	9.13517	1.99E − 05
SEC24D	0	0.277004	0.561383	0.038512	6.92448	2.41E − 05
P3H1	0	0.245535	0.768787	0.040094	6.543159	2.82E − 05
CCND1	0	0.109714	0.61351	0.043593	9.025389	3.77E − 05
GMPR	0	0.328295	1.158899	0.043593	3.489729	4.32E − 05
HLA‐DRA	0	−0.09297	−0.87987	0.043593	8.289285	3.74E − 05
NPY2R	0	−0.37247	−1.21761	0.043593	5.112414	4.43E − 05
COL4A6	0	−0.3962	−1.1545	0.047131	4.526117	6.47E − 05
FILIP1L	0	0.032205	0.8006	0.047131	5.555825	5.39E − 05
LOXL2	0	0.335066	1.176323	0.047131	4.564733	6.63E − 05
P4HA2	0	0.164117	0.920069	0.047131	6.167042	6.19E − 05
PCDHGC5	0	0.522863	0.859332	0.047131	6.510753	6.17E − 05
DHX35	0	4.368268	0.246487	0.048523	−3.06674	7.76E − 05
FKBP14	0	0.133249	0.62127	0.048523	5.325963	7.8E − 05
GSN	0	0.401608	0.743679	0.048523	8.535698	7.97E − 05

With the significance denoted by FDR < 0.05, 21 genes are identified as significantly changed (Table 1). The heatmap diagram illustrates the differences in the expression of these genes between the samples of the three groups (Figure 2d). The genes can be considered the ones that are generally altered by lithium consumption. In the following steps, each of the low and high doses of the drug was compared with the control separately to study the therapeutic and chronic effects of Li on the BPD hippocampal transcriptome.

### 3.4 Gene expression profiles in the therapeutic dose of lithium

**FIGURE 3:**
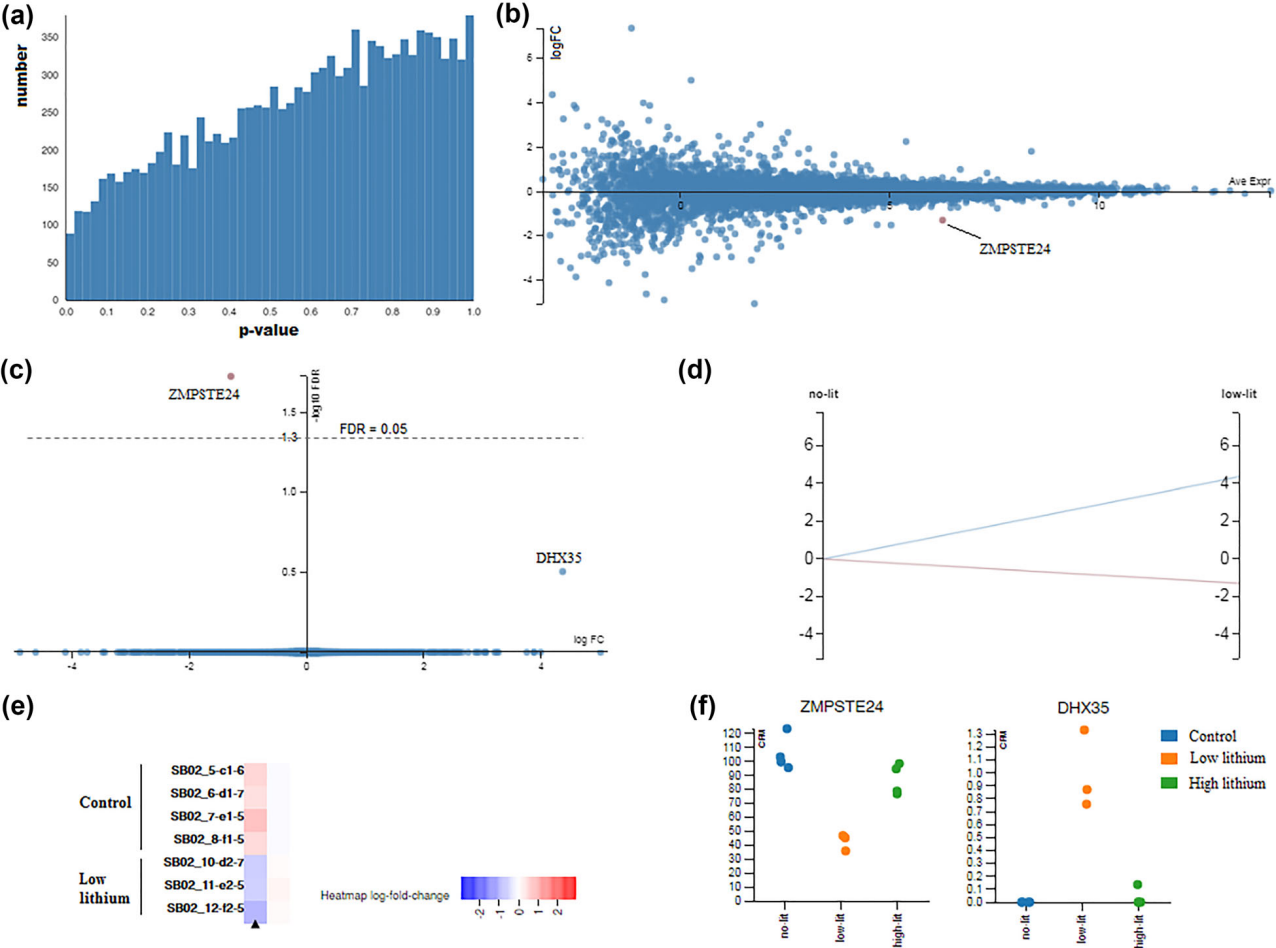
Low‐dose lithium treatment compared with control: (a) *p*‐value histogram; (b) MA plot (logFC vs. average expression). Red points indicate the genes with significant expression change; (c) volcano plot for the low‐dose Li compared with control; (d) parallel coordinates diagram; (e) heatmap for the genes important in the low‐dose/control comparison; (f) count per million (CPM) of the genes ZMPSTE24 and DHX35 for the samples in the study groups.

The expression profiles were compared between control and low‐dose lithium treatments. This analysis can represent the comparison of a drug‐treated progenitor cell in the hippocampus with an untreated one, and it shows what genes are subject to altered expression in the therapeutic doses of lithium. The frequency distribution of *p*‐values for this comparison has been illustrated in Figure 3a.

MA plot (M denoting log ratio, vs. A denoting average expression) is a graph used for pairwise comparisons and shows logFC versus the average expression of genes. Genes with significantly changed expression are displayed with red dots. Genes with low expression (leftward *x*‐axis) show a wider, but more erroneous, range of changes, and are characteristically insignificant. Significant genes typically appear in the middle or the right end of the *x*‐axis (Figure 3b).

The volcano plot compares two groups by showing the logarithmic significance of the expression changes versus logFC. The amount and significance of the expression change can be presented visually using this graph. With FDR < 0.99, the two genes, ZMPSTE24 and DHX35, were recognized as having the highest expression changes (Figure 3c). The expression change of ZMPSTE24 was significant (Table 2).

**TABLE 2:** Genes undergoing the highest change of expression in the therapeutic dose of Li, compared to the control.

Gene	No‐lit	Low‐lit	FDR	AveExpr	*p* Value
ZMPSTE24	0	−1.29236	0.018804	6.2574	1.46E − 06
DHX35	0	4.368343	0.517904	−3.06679	8.02E − 05

Figure 3d,e depicts the parallel coordinates and heatmap diagrams for the gene expression change of low‐dose samples in comparison with the control. Read counts for each of ZMPSTE24 and DHX35 in the three studied samples have also been plotted in Figure 3f. The decrease or increase in the expression of the two genes at low doses compared to the control can be observed in the graphs.

Considering that the low dose of lithium is the usual dose in treating BPD, the above analyses actually show what genes are changed in the therapeutic doses of lithium. Therefore, ZMPSTE24 and DHX35 can be introduced as important genes in lithium therapy.

### 3.5 Gene expression alterations with chronic lithium treatment

**FIGURE 4:**
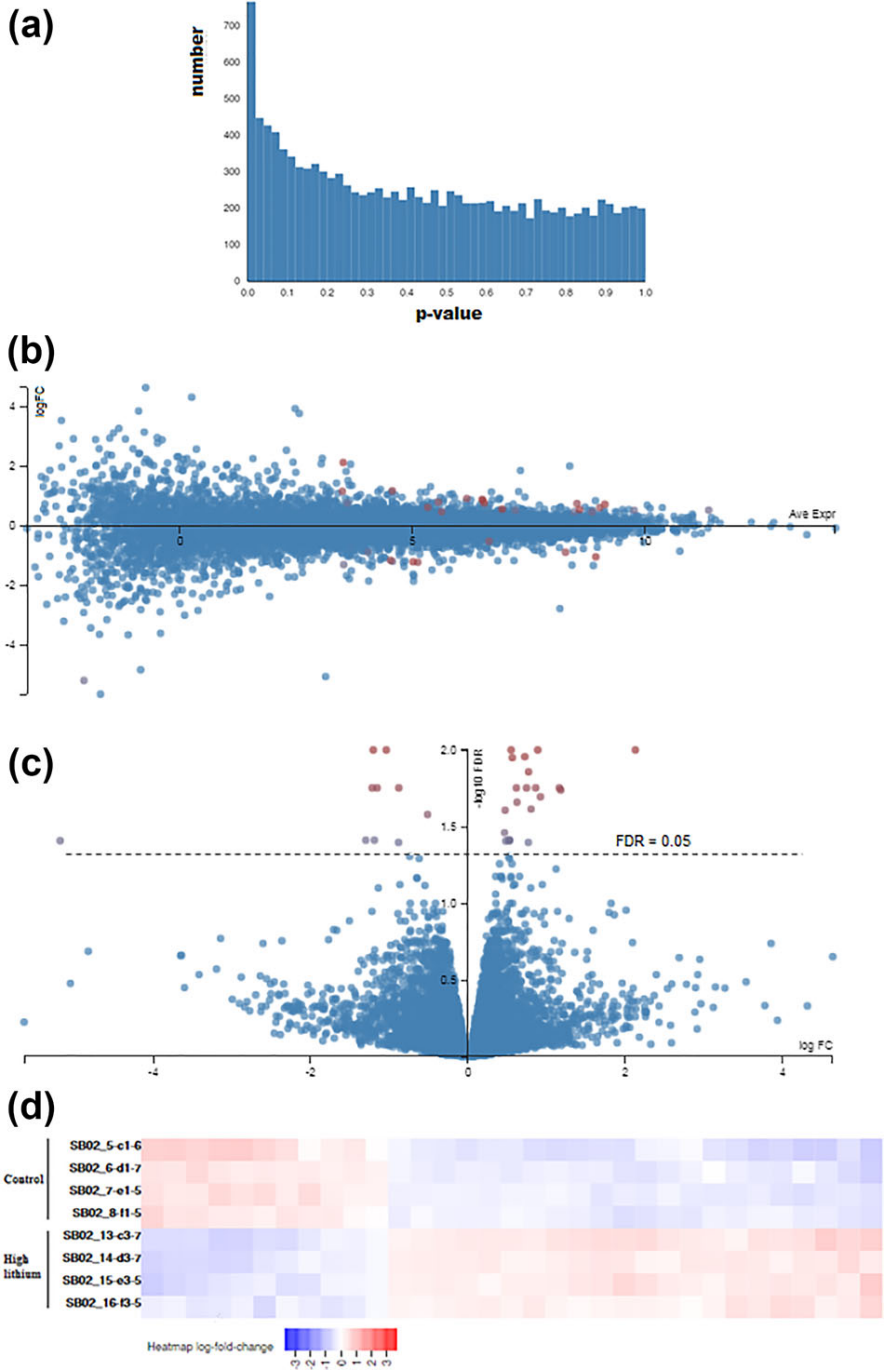
High‐dose lithium treatment compared with control: (a) *p*‐value histogram; (b) MA plot (logFC vs. average expression). Red points indicate the genes with significant expression change; (c) volcano plot for the high‐dose Li compared with control; (d) heatmap for the genes with significantly changed expression in the high‐dose/control comparison.

The expression profiles were compared between the control and the samples treated with high‐dose lithium to emulate the chronic effects of Li on the hippocampal progenitor cells. Figure 4a shows the frequency distribution of *p*‐values in this pairwise comparison. Figure 4b illustrates the MA plot and the significant genes (in red). In Figure 4c, the volcano plot for the gene expressions compared between the control and the high‐dose sample is depicted.

At the significance level of FDR < 0.05, 33 genes demonstrate expression different from the control sample, of which 11 genes have decreased expression, and 22 have increased expression in the presence of lithium (Table 3). The heatmap diagram (Figure 4d) shows how the expression of these genes changes (whether increasing or decreasing) compared to the control.

**TABLE 3:** Genes undergoing a significant change of expression at the high (chronic) dose of Li, compared to the control.

Gene	Control	High lithium	FDR	AveExpr	*p* Value
CD74	0	−1.03943	0.009864	8.941747	3.86E − 06
HLA‐DMA	0	−1.20494	0.009864	5.011998	1.23E − 06
L1CAM	0	2.125241	0.009864	3.508505	2.57E − 06
PDLIM1	0	0.885089	0.009864	6.493979	2.33E − 06
SSR3	0	0.546547	0.009864	8.591011	3.12E − 06
PMEPA1	0	0.721328	0.010912	9.13517	5.12E − 06
SEC24D	0	0.561383	0.01108	6.92448	6.06E − 06
P3H1	0	0.768787	0.013718	6.543159	8.58E − 06
CCND1	0	0.61351	0.017473	9.025389	1.58E − 05
COL4A6	0	−1.1545	0.017473	4.526117	1.83E − 05
GMPR	0	1.158899	0.017473	3.489729	1.49E − 05
GSN	0	0.743679	0.017473	8.535698	2.05E − 05
HLA‐DRA	0	−0.87987	0.017473	8.289285	1.95E − 05
NPY2R	0	−1.21761	0.017473	5.112414	1.32E − 05
PCDHGC5	0	0.859332	0.017473	6.510753	1.64E − 05
LOXL2	0	1.176323	0.018043	4.564733	2.26E − 05
P4HA2	0	0.920069	0.019991	6.167042	2.66E − 05
FKBP14	0	0.62127	0.021695	5.325963	3.05E − 05
FILIP1L	0	0.8006	0.024049	5.555825	3.57E − 05
LARGE1	0	0.470488	0.02446	5.623768	3.82E − 05
IVD	0	−0.51461	0.026088	6.643558	4.28E − 05
CAP1	0	0.463818	0.034321	8.864742	5.9E − 05
HLA‐DMB	0	−1.30026	0.038384	3.517863	7.8E − 05
HLA‐DOA	0	−1.19045	0.038384	4.568304	7.2E − 05
SLC39A14	0	0.518467	0.038384	7.209742	7.65E − 05
SPARC	0	0.527818	0.038384	11.36906	7.25E − 05
CALU	0	0.520565	0.03871	9.76633	8.31E − 05
SIX5	0	−5.18328	0.03871	−2.06699	8.47E − 05
CEMIP2	0	0.477828	0.038867	8.471255	8.81E − 05
ACOT8	0	0.765674	0.039765	3.598541	9.64E − 05
BBS1	0	−0.88256	0.039765	4.039036	9.55E − 05
ARSG	0	−0.74045	0.04909	3.675753	0.000123
PRNP	0	0.516133	0.04909	6.622134	0.000127

### 3.6 Gene interaction network and pharmacogenomics

As it was shown, in the usual dose of lithium treatment, ZMPSTE24 and DHX35 demonstrated considerably changed expression. In high doses, 33 genes underwent altered expression (including ZMPSTE24 and DHX35), which represent the lithium response genes. With the 2 genes set aside, the remaining 31 genes can be considered involved in lithium's effects on the hippocampus tissue.

**FIGURE 5:**
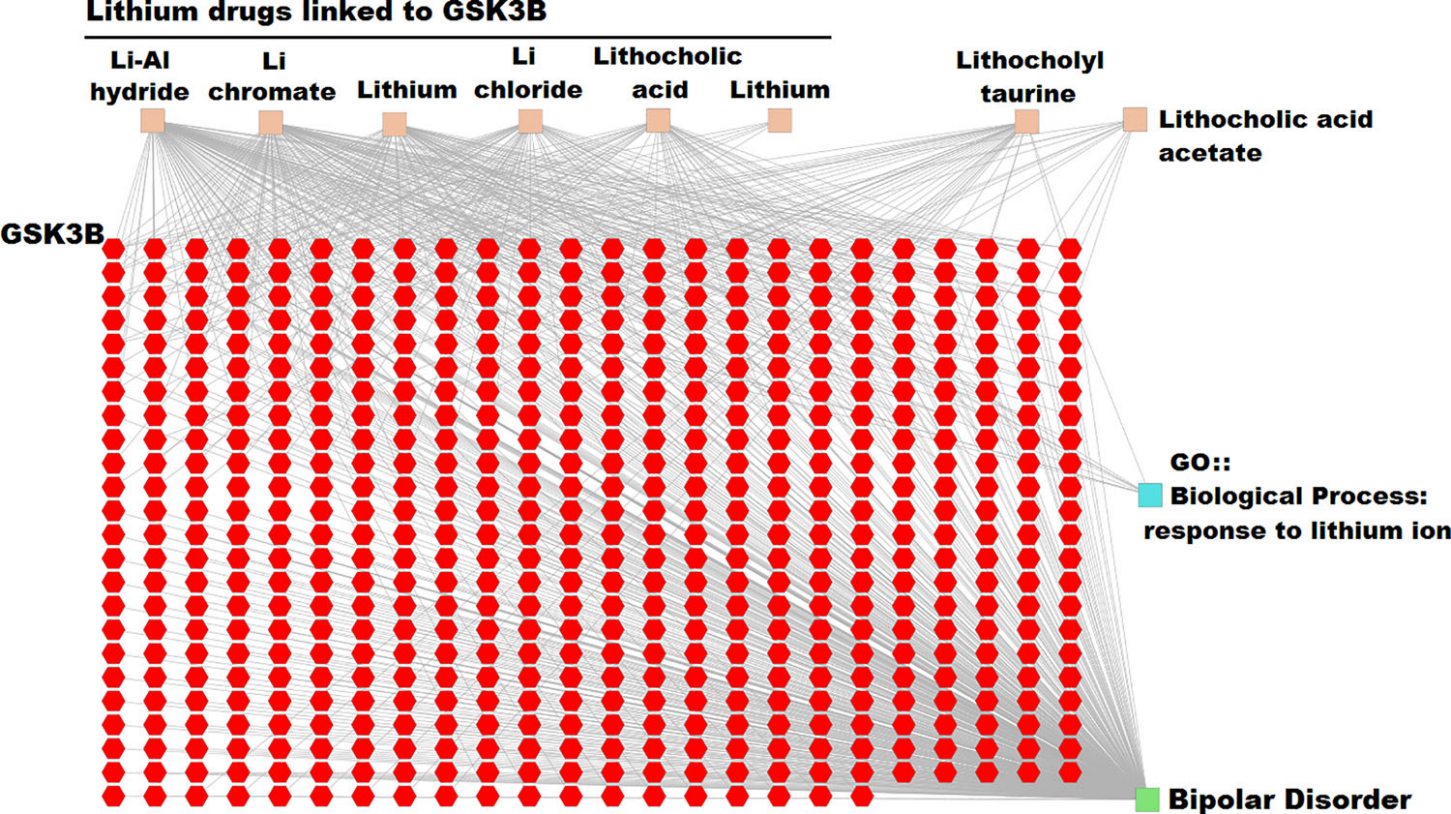
Pharmacogenomic network of lithium in hippocampal progenitor cells after chronic lithium treatment. Red points indicate the genes.

Examining the gene‐disease relationship indicated 1461 genes related to BPD. Next, the disease‐related genes in hippocampal tissue (1461) and our identified lithium response genes (33) underwent enrichment analysis. That is, their relationship with biological functions, diseases, and drug responses was investigated. Only genes with significant relationships (Bonferroni‐adjusted *p*‐value <.05) were considered, which comprised 571 genes. The network map of these genes was drawn and analyzed, depicted in Figure 5.

To extract pharmacogenomic information, we first examined the intersection of disease‐related genes in the hippocampus (1461 genes) and the genes registered in databases as lithium response genes. Using the node degrees as the criterion, 14 genes (Table 4) were identified as the main players in the pharmacogenomics of Li in the hippocampus of BPD brains. These genes are involved both in the BPD pathogenesis and in the lithium's drug response in the hippocampal tissue.

**TABLE 4:** Bipolar disorder‐related genes linked to drug response in lithium therapy.

Gene	Protein	Degree
GSK‐3β	Glycogen synthase kinase 3 beta	8
AKT1	AKT serine/threonine kinase 1	7
GSK‐3α	Glycogen synthase kinase 3 alpha	7
AVP	Arginine vasopressin	6
BDNF	Brain derived neurotrophic factor	6
CALR	Calreticulin	6
CTNNB1	Catenin beta 1	6
CXCL8	C‐X‐C motif chemokine ligand 8	6
DRD2	Dopamine receptor D2	6
GRIN2A	Glutamate ionotropic receptor NMDA type subunit 2A	6
IL‐6	Interleukin 6	6
IMPA1	Inositol monophosphatase 1	6
MAPK1	Mitogen‐activated protein kinase 1	6
TNF	Tumor necrosis factor	6

Gene ontology analysis, which relates genes to biological processes, was also performed, and genes related to the lithium response processes were identified. This analysis showed that many genes that are activated or deactivated under the influence of lithium drugs play a known role in growth and signaling pathways related to cell proliferation (Table 5).

**TABLE 5:** Genes linked to the biological process ontology of “response to lithium ion.”

Gene	Protein
FAS	Fas cell surface death receptor
TPH2	Tryptophan hydroxylase 2
ADCY1	Adenylate cyclase 1
IGFBP2	Insulin like growth factor binding protein 2
CALR	Calreticulin
PPARG	Peroxisome proliferator activated receptor gamma
GSK‐3α	Glycogen synthase kinase 3 alpha
NFATC3	Nuclear factor of activated T cells 3
NFE2L2	Nuclear factor, erythroid 2 like 2

In order to identify the genes involved in the hippocampal effect of lithium, we investigated the common cases between the disease‐related genes in the hippocampal tissue (1461) and the identified lithium response genes (33). As a result, three genes, including CCND1, LOXL2, and PRNP, were identified as the ones related to Li's chronic effects in BPD patients.

## 4 DISCUSSION

Network pharmacology attempts to establish a relationship between medicine and disease through targets. Applying the systems approach to investigate this relationship allows for discovering all possible drug targets and relevant beneficial or adverse effects simultaneously (Sharma, Jadhav, et al., 2022; Sharma, Singla, et al., 2022).

Lithium has been used as the first‐line treatment for BPD patients for decades. Genomic aspects of the Li mechanism and its pharmacological response have been investigated by Picard (Pickard, 2017). The role of genetics in response to lithium in bipolar patients has been broadly reviewed (Papiol et al., 2018; Pisanu et al., 2018; Rybakowski, 2014). Moreover, gene variants associated with lithium drug response in BPD have been identified through GWAS and pharmacogenomics studies (Hou et al., 2016). However, some side complications of this drug are still elusive. The use of lithium in BPD patients, especially in the long term, affects the hippocampus and causes it to enlarge (Hajek et al., 2012; Hibar et al., 2016). This is despite the fact that psychiatric abnormalities are generally associated with decreased hippocampal volume (Hibar et al., 2016). Therefore, it seems that Li may protect existing nerve cells or stimulate neurogenesis in the hippocampus. This study aimed to investigate the effect of lithium on the enlargement of the hippocampal volume in BPD patients by using systems pharmacology to identify the genes involved in the hippocampal size change and the possible mechanisms of this phenomenon, by analyzing the transcriptome data.

Unlike other mammalian cerebrospinal organs, the hippocampus is able to generate neurons even at puberty; about 700 new neurons are produced per day in the human dentate gyrus (Spalding et al., 2013; Stangl & Thuret, 2009). The new cells differentiate into neuroblasts, neurons, or microglia. Accordingly, all genes involved in growth, proliferation, and cell differentiation pathways can be candidates for the increase in the hippocampal volume. By gathering, comparing and analyzing the genes contributing to the BPD pathogenesis and the lithium drug response genes, this study attempted to find the genes involved in increased hippocampal volume. This analysis identified three genes as the main candidates for this phenomenon, including CCND1, LOXL2, and PRNP. CCND1 encodes the protein Cyclin D1, which is highly expressed in the brain and is associated with the occurrence of neurological and behavioral phenotypes (Abdullah et al., 2007). The next identified gene, LOXL2, encodes lysyl oxidase like 2. This protein is involved in the deamination of lysine, and its deficiency has been observed as a characteristic of neurological diseases. LOXL2 mutations have been shown to cause abnormal growth of bulges or ballooning in cerebral vasculature (Wu et al., 2018). The third gene, PRNP, encodes the human prion protein, a well‐known factor whose misfolding and aggregation leads to its important contribution to brain and nerve tissue diseases, including BPD (Weis et al., 2008). Based on these findings, the genes CCND1, LOXL2, and PRNP are suggested to be linked with neuropathologies or cell growth in the brain, indicating their likely contributions to chronic lithium effects.

Network analysis and gene set enrichment identified GSK‐3β, the main target of lithium, as one of the most important proteins contributing to the drug response of Li, as confirmed in other studies (Iwahashi et al., 2014). This enzyme catalyzes the inactivating phosphorylation of glycogen synthase and is involved in energy metabolism and neuronal cell development (De Falco et al., 2022). In fact, BPD is often associated with impaired energy metabolism and mitochondrial function, and there is ample evidence of symptoms of psychosis and emotional and cognitive decline present in mitochondrial disorders (Clay et al., 2011). Therefore, GSK‐3β may be an important mediator of the abnormal growth of the hippocampus in BPD patients.

Additional analyses in this study, that is, the comparison of gene expression in control and low (therapeutic) dose lithium samples, introduced ZMPSTE24 and DHX35 as important genes in lithium therapy. ZMPSTE24 encodes FACE1 protein, which is a zinc‐dependent metalloprotease acting on lamin A/C. Its main role is in adipogenesis, and its deficiency has been observed in some dystrophies and dysplasias (Quigley et al., 2013); however, its function in the nervous system is not clear, requiring more research. DHX35 encodes a protein of the same name, which plays a role in RNA processing and its preparation for translation and splicing. This gene is also related to dysplasia and is believed to play a role in cell growth and division, but its exact function remains unknown (Xu et al., 2002).

An aspect of limitation in this study is the conclusion based on in vitro experiments; accordingly, in vivo tests are recommended to address this point. Second, the choice of 2.25 mM as the high‐dose exceeds the recently reported maximum hippocampal Li concentration of 1.4 mM 2020. However, the doses applied here demonstrated overlapping molecular effects in response to the low‐ and high‐dose conditions (Palmos et al., 2021); therefore, the results obtained using the selected dosing can actually encompass and represent the effects of this maximal physiological concentration. Though the possible toxic effects of the high dose in patients are acknowledged, our results can show a valid picture of lithium dose effects at the cellular level as they have shown no effect on hippocampal progenitor cells’ proliferation and death (Palmos et al., 2021). As an additional point, regardless of what the real physiological Li concentration is, directly extrapolating drug doses from in vivo to in vitro experiments comes with its own complications (Yoon et al., 2012).

## 5 CONCLUSION

Modulated expression of CCND1, LOXL2, and PRNP genes was shown to mediate the complicated growth and differentiation of human hippocampal progenitor cells. From the mentioned findings and previous literature, it can be concluded that lithium may increase the size of the hippocampus in bipolar patients by promoting the generation of new neurons and stimulating their differentiation into neuroblasts, neurons, or microglia. For future work, conducting molecular genetic studies to verify the role of these genes and applying lithium treatments to confirm its enhancing effect on the genes using specific probes are warranted.

## CONFLICT OF INTEREST STATEMENT

The authors declare no conflicts of interest.

### PEER REVIEW

The peer review history for this article is available at https://publons.com/publon/10.1002/brb3.3215.

## Data Availability

The data that support the findings of this study are available from the corresponding author upon reasonable request.
